# The courses of maternal and paternal depressive and anxiety symptoms during the prenatal period in the FinnBrain Birth Cohort study

**DOI:** 10.1371/journal.pone.0207856

**Published:** 2018-12-17

**Authors:** Riikka Korja, Saara Nolvi, Eeva-Leena Kataja, Noora Scheinin, Niina Junttila, Henna Lahtinen, Suoma Saarni, Linnea Karlsson, Hasse Karlsson

**Affiliations:** 1 University of Turku, Institute of Clinical Medicine, Turku Brain and Mind Center, FinnBrain Birth Cohort Study, Turku, Finland; 2 University of Turku, Department of Psychology, Turku, Finland; 3 University of Turku and Turku University Hospital, Department of Psychiatry, Turku, Finland; 4 University of Turku, Department of Teacher Education, Turku, Finland; 5 Helsinki University Hospital, Department of Psychiatry, Helsinki, Finland; 6 University of Turku and Turku University Hospital, Institute of Clinical Medicine, Department of Child Psychiatry, Turku, Finland; Chiba Daigaku, JAPAN

## Abstract

Maternal prenatal symptoms of depression and anxiety have been suggested to impose differential effects on later offspring development, depending on their characteristics, such as timing, intensity and persistence. Paternal symptoms have been less investigated. While knowledge on these trajectory characteristics is essential for improved comprehension of prenatal stress, prospective studies including both expecting parents have been scarce. We aim at identifying and comparing the trajectories of prenatal depressive and anxiety symptoms in both parents in a pregnancy cohort design. The sample included 3202 mothers and 2076 fathers who were recruited to the FinnBrain Birth Cohort study (www.finnbrain.fi). Depressive symptoms were assessed using the Edinburgh Postnatal Depression Scale (EPDS) and general anxiety by the anxiety scale of the Symptom Checklist -90 (SCL-90) repeatedly at 14, 24, and 34 gestational weeks. Five differential depressive and four anxiety symptom trajectories were identified across pregnancy both in mothers and in fathers. The trajectories of consistently low depressive or anxiety symptoms were associated with higher educational level in both parents, and with nulliparity and non-smoking during pregnancy in mothers. Parents with consistently high or increasing levels of symptoms had more often prenatal SSRI medication. The congruences between elevated depressive and anxiety symptoms at any point in pregnancy, as well as parental trajectories within families were low. However, in this population-based sample, the self-reported symptom levels of both parents were generally very low. Variance in timing and persistence of parent-reported prenatal depressive and anxiety symptoms is potentially important, while symptom trajectories are very similar in mothers and fathers. These differential symptom trajectories and the significance of their correlates should be acknowledged when studying prenatal stress exposures and the related outcomes in children.

## Introduction

Maternal psychiatric symptoms during pregnancy reportedly have diverse and often adverse influences on child development, and ultimately on offspring adult health [1–5]. Paternal symptoms during the pre- and postnatal period and their associations with offspring development have been investigated to a lesser extent [6–8]. Depression and anxiety are the most common psychiatric symptoms during the prenatal and early postnatal periods both in women as well as men. Women commonly present with more pronounced depressive and anxiety symptomatology than men, also during pregnancy [9,10]. Clinically significant prenatal depressive symptoms affect approximately 10–20% of mothers and 2–13% of fathers [8,10–16]. The prevalence of prenatal anxiety symptoms in different studies varies from 9.5% to 29% in mothers and from 8% to 14% in fathers [9,10,15,17,18]. The variation in prevalence estimates is partly explained by the timing of the measurement and the method of assessment. Many background factors, such as history of previous psychiatric disorders, low socioeconomic status and pregnancy-related somatic risk factors increase the risk for prenatal depression or anxiety [19,20]. Comorbidity between depression and anxiety has shown to be high also during pregnancy [15,19,21]. However, depression and anxiety are also partially distinct conditions, supported by the notions of differential symptom course trajectories [22], clinical prognostic factors [22,23] and pathophysiology [24]. Furthermore, previous evidence suggests that prenatal anxiety and depressive symptoms may also have differences in their biological correlates [25] as well as distinct effects on fetal development [2,26]. Therefore, parental anxiety and depression should be studied independently, especially in the context of prenatal fetal exposures.

Previous reports on the longitudinal course of parental prenatal depressive and anxiety symptoms comprising both parents have been scarce and inconsistent. In several studies, depressive symptoms have shown to decline throughout the prenatal period and onwards, both in mothers and in fathers [9,10] However, one recent study reported highest levels of depressive symptoms during the first and third trimesters, in mothers [27]. Similarly, prenatal anxiety symptoms have shown to follow a U-shaped pattern with highest levels of symptoms during the first and third trimesters in both parents [9,10,27]. On the other hand, some studies have reported the peak in paternal anxiety symptoms to occur at mid-pregnancy, where after anxiety would decrease steadily and across the postpartum period [28,29]. As a result of these discrepancies, it has been suggested that the timing and duration of depressive and anxiety symptoms in the prenatal period do not follow a uniform course, implying considerable heterogeneity in symptoms between individuals [30,31]. This has important implications for the concept of prenatal stress exposure and for studies investigating offspring outcomes related to parental symptoms. By studying symptom trajectories, more detailed information on the individual differences in timing, intensity and consistency of parental symptoms could be identified.

Parental mental health trajectories have previously been studied mainly during the transition from the third trimester of pregnancy to postpartum or from the perinatal period into childhood years [32–36]. Studies based on general population samples concur in that most parents report only minimal or mild symptoms across the prenatal period and postpartum [33–37]. In addition, most studies have identified a group of continuously high depressive or anxiety symptom levels [32–37]. These studies have mainly included only one prenatal symptom assessment, usually during the third trimester [8,32,34–36]. However, the importance of investigating the courses of depressive and anxiety symptoms across trimesters has been highlighted in several reports on the negative effects of prenatal psychiatric symptoms on child development and well-being [5,38]. Identifying differential courses of symptoms during pregnancy may provide a basis for a more detailed investigation of diverse offspring outcomes, related to variation in timing and chronicity of exposure to prenatal psychological distress.

Despite the strong evidence that fathers have a crucial role in child well-being [39], the research on parental mental health trajectories has mainly focused on mothers. A few studies have suggested that elevated paternal depressive or anxiety symptoms during transition from the third pregnancy trimester to the postpartum period increase the risk for later problems in parenting [26] and child socio-emotional development [7,8]. The mechanisms behind the possible effects of maternal and paternal psychiatric symptoms on child’s outcome are at least partially different [8,40,41]. Maternal depression or anxiety during pregnancy has suggested to affect the child mainly through different intrauterine processes, including HPA-axis activity, functioning of the placenta, changes in the immunological milieu, or nutritional supply [38,42,43]. Instead, the effects of paternal psychiatric symptoms on the child may either be mediated by the mother experiencing higher psychosocial distress due to the father’s psychological distress [40,41] or by potential epigenetic effects of in paternal sperm, resulting in DNA methylation in the offspring [44,45]. Moreover, direct genetic effects are among the potential mechanisms to explain the effects of prenatal distress on child outcomes, for both parents, as child characteristics and prenatal distress experienced by the parents may indicate a shared underlying genotype [46,47]. Overall, very few studies focus on paternal and maternal prenatal psychiatric symptoms simultaneously within the same study. In addition, as far as we know, there are no studies on the trajectories of paternal or spousal psychiatric symptoms covering most of the prenatal period by repeated prospective measurements.

The aim of this study was to analyze the trajectories of prenatal depressive and anxiety symptoms and the associations of these trajectories with sociodemographic factors in both mothers and fathers in a population-based pregnancy cohort. In addition, the aim was to evaluate the congruence of the anxiety and depression trajectories and the congruence between paternal and maternal symptom trajectories inside the families. To our knowledge, this is the first study evaluating the symptom trajectories across trimesters in both mothers and fathers. The study is part of the large FinnBrain Birth Cohort Study that prospectively investigates the long-term effects of early life stress exposures on e.g. brain development.

## Methods

### Sample

Recruitment for the FinnBrain Birth Cohort Study took place at maternal welfare clinics between December 2011 and April 2015 in the South-Western Hospital District and the Åland Islands in Finland. The study subjects gave their written informed consent. the Ethics Committee of the Hospital District of Southwest Finland approved the study protocol 14^th^ June 2011. The study population (N = 3808 families) comprises of women that attended the free-of-charge ultrasound at the gestational week 12, their children-to-be-born and fathers of the children/partners of the mothers [48]. Verified pregnancy and sufficient knowledge of either Finnish or Swedish (the official languages in Finland) were required for participation. Of those informed about the study (N = 5790), a total of N = 3808 mothers and N = 2623 fathers or other partners of the mothers decided to participate. In this study, only reported fathers of the children participating were included, and other partners of mothers in the study were excluded. Mothers (N = 3202) and fathers of the children (N = 2076) responding to at least one symptom questionnaire during pregnancy were included.

### Procedure and measures

After recruitment, the participants filled in a set of self-report questionnaires three times during pregnancy; at gestational weeks (gwks) 14, 24, and 34. The questionnaires comprised of standardized and internationally validated measures and they could be filled in online or sent via postal mail. Prenatal depressive symptoms were evaluated in both parents using the Edinburgh Postnatal Depression Scale (EPDS, 34) at gwks 14, 24 and 34. EPDS is a self-report questionnaire consisting of 10 items. The parent is asked whether she or he has experienced the proposed symptoms during the last two weeks using a 4-point Likert scale. The EPDS has been studied extensively and it is thought to be a valid screen for both pre- and postnatal depression [49–53], also in fathers [54,55]. Prenatal anxiety of both parents was evaluated using the anxiety scale of Symptom Checklist 90 (SCL-90) at gwks 14, 24 and 34. The anxiety subscale of SCL-90 is a reliable and valid measure of anxiety symptoms in both clinical and research settings [56] and consists of 10 items rated from 0 to 5.

The sum scores of the EPDS and SCL-90 anxiety scales were used as continuous variables in the data analyses and they demonstrated good internal consistency (Cronbach’s α’s ranging from .82 to .84 in mothers and .79 to .81 in fathers for EPDS and .83 to .85 in mothers and .83 to .86 in fathers for SCL-90). The background variables i.e. relationship status, educational level, age, parity, and paternal use of serotonin reuptake inhibitors (SSRIs) during the index pregnancy were drawn from the questionnaires filled in at gwk 14. Educational level was categorized into four classes: secondary school (less than 9 years of schooling), high school/vocational education, university/polytechnics degree, or higher (doctoral/licenciate). No family income variable was available. Therefore, only variables describing maternal and paternal education were used as indicators of socioeconomic status. Other background variables i.e. maternal prenatal smoking, the household variable (living together) and maternal use of serotonin reuptake inhibitors (SSRIs) during the index pregnancy, were drawn from the questionnaires filled in at 34 gwks. In all, 80% of the mothers using SSRI at 34 gwks had already used SSRI-medication at 14 gwks. Father’s use of SSRI was measured only at 14 gwks.

### The validity of the measures

The measurement validities of EPDS and SCL-90 anxiety subscale were first examined using confirmatory analysis (CFA) that tests the adequacy of the specified relations, whereby indicators are linked to their underlying latent constructs [57]. The following indices indicating fit of the models were used: the Chi-square test, the Root Mean Square Error of Approximation (RMSEA, values close to .06 indicating a good fit), the comparative fit index (CFI, values close to .90 indicating a suitable model), and the standardized root mean square residual (SRMR, values below .08 reflecting good fit) (see [58,59]). Concerning EPDS, the fit indices were acceptable i.e. χ^2^ (df) = 1781.46 (366), p < .001, CFI = .94, RMSEA = .04, SRMR = .04 for maternal data and χ^2^ (df) = 894.23 (366), p < .001, CFI = .95, RMSEA = .03, SRMR = .04 for paternal data. Concerning SCL-90 models, the errors of the verbally similar items 5 and 10; 7 and 10 and consecutive items 9 and 10 were allowed to correlate. After this, the fits of the models were acceptable: χ^2^ (df) = 1667.42 (366), p < .001, CFI = .93, RMSEA = .03, SRMR = .04 for maternal data and χ^2^ (df) = 1291.005 (366), p < .001, CFI = .90, RMSEA = .04, SRMR = .05 for paternal data.

### Statistical analyses

The associations between the means of depressive and anxiety symptom scales at each time point in mothers and in fathers were investigated with zero-order Spearman correlations. As measures of effect sizes, Cohen’s d and partial ƞ^2^ were used. Structural Equation Modeling in Mplus 6, and latent growth mixture modeling, were conducted to examine the trajectories of maternal and paternal EPDS and SCL-90 symptoms [60]. Latent growth mixture modeling (LGMM) is a method for identifying multiple unobserved sub-populations (latent classes), describing the longitudinal change within each latent class and examining differences in the change among the latent classes [61]. The numbers of latent classes consisting of mothers’ and fathers’ EPDS and SCL-90 scores were determined by increasing the numbers of classes in separate analyses and observing the change in indices. The indices that were used in decision-making were Bayesian Information Criterion (BIC), Akaike Information Criterion (AIC) [60,62], Entropy value (with values closer to 1.0 indicating higher confidence of classification; [63] and posterior probabilities of class membership. Furthermore, the theoretical and clinical interpretability of the class solutions were used as a help in choosing the best model, a step considered necessary in the decision-making in LGMM [64]. Missing values were treated with the Expectation Maximization (EM) method in Mplus. The reliabilities of the final solutions were checked by replicating the analyses with non-imputed datasets (consisting complete data from 2157 mothers and 1475 fathers). The resultant model solutions (with and without EM) were alike. Next, the associations between trajectories and parental age, education, parity and SSRI medication use were investigated using ANOVA (age) and cross-tabulation with χ^2^ test (education, parity and SSRIs).

## Results

### Demographics and attrition

Parents with missing questionnaire data over the course of pregnancy had lower educational levels (χ^2^ (3) = 38.76, p < .001) and were more often single or divorced (χ^2^ (1) = 22.26, p < .001) than those who filled in the questionnaires at each of the three assessment points. No differences were seen in age, parity or in the level of depressive or anxiety symptoms at 14 gwks between those parents who filled in all questionnaires and the parents with missing questionnaire data. In the study sample, the mothers had higher educational levels and they were younger than the fathers. In addition, mothers reported more depressive and anxiety symptoms at each assessment point compared to fathers. Descriptive statistics and comparisons between mothers and fathers are displayed in Table 1. Maternal depressive (continuous variables) and anxiety symptoms (continuous variables) were intercorrelated at all assessment points during pregnancy (r = .58-.62, p < .001). Similarly, paternal depressive and anxiety symptoms were intercorrelated at all assessment points (r = .58-.59, p < .001).

**Table 1:** The descriptive statistics on background and study variables in the subgroups of mothers and fathers. P values and Cohen’s d’s are given for the differences between mothers and fathers.

	Mothers	Fathers		
	N = 3202	N = 2076		
N (total) = 5278	Mean (SD)	Mean (SD)	p<	Cohen's d ****
Age	30.92 (4.56)	32.60 (5.33)	.001*	
Marital status (%)				
In relationship	90.9	89.9		
Missing	7.6	9.7		
Parents living together	93.0			
Education (%)				
Primary school	2.9	4.6		
Secondary	33.1	41.2		
University	55.2	46.2		
Higher	4.1	2.6		
**Missing**	4.7	5.4		
Parity (%)				
Nulliparous	49.1	NA		
Multiparous	45.9	NA		
Missing	5.1	NA		
SSRIs medication (%)	3.1#	2.1##		
Smoking	14.2#			
Anxiety (SCL)				
14 gwks	3.31 (3.91)	2.45 (3.48)	.001***	0.22
24 gwks	3.92 (4.26)	2.66 (3.81)	.001***	0.31
34 gwks	3.24 (4.00)	1.94 (3.20)	.001***	0.36
Depression (EPDS)				
14 gwks	5.17 (4.02)	3.74 (3.42)	.001***	0.38
24 gwks	5.00 (4.12)	3.50 (3.47)	.001***	0.40
34 gwks	4.91 (4.10)	3.13 (3.38)	.001***	0.47

* t-test

** χ^2^ -test

***logistic regression

**** , #3^rd^ pregnancy trimester

## 1^st^ pregnancy trimester

Cohen`s d effect size d > .20 = small; d > .50 = medium; d > .80 = large.

### The trajectories of maternal and paternal prenatal depressive and anxiety symptoms

**Fig 1:**
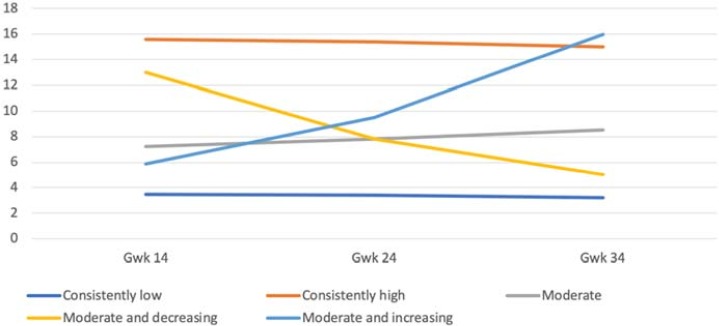
The five-class solution of the trajectories of maternal depressive symptoms during pregnancy.

Different class solutions were examined to obtain the best fit of the model for maternal and paternal prenatal depressive and anxiety symptoms. Regarding *maternal depressive symptoms* the four- and five-class-solutions resembled each other in terms of statistical indices, but the five-class solution had a better fit with the assumption of trajectories and it was thus chosen (Fig 1). The resultant fit indices are presented in Table 2. The five-class solution yielded a large group of mothers (N = 2157, 67%) with consistently low and only slightly decreasing symptoms throughout pregnancy (M = 3.40; Estimate = –0.31, p < .001). Estimate refers to the change in symptoms in each group over the course of pregnancy. Two smaller groups were extracted: mothers (N = 67; 2%) who had consistently high (M = 15.86; Estimate = –0.81, p = .11) symptom levels and a group of mothers (N = 72, 2%) with moderate and increasing symptoms (M = 5.67; Estimate = 4.92, p < .001). The fourth group (N = 773, 24%) had moderate and relatively stable symptoms (M = 7.37; Estimate = 0.67, p < .01). The fifth group (N = 131, 4%) had an initially high symptom score indicating a potentially clinically relevant level of symptoms (M = 12.44) in early pregnancy, but the level of symptoms decreased to a subthreshold level (Estimate = –3.96, p < .001) towards the end of pregnancy.

**Table 2:** The growth mixture model indices: Trajectories of maternal depressive symptoms during pregnancy.

	AIC	BIC	Entropy	Class Proportions	Average Latent Class Posterior Probabilities
EPDS					
1 Class	44516.29	44564.85	1.000	1.000	1.000
2 Class	44161.04	44227.82	.73	.16/.84	.83/.94
3 Class	43908.77	43993.76	.77	.83/.08/.09	.93/.78/.78
4 Class	43765.96	43869.15	.79	.74/.05/.02/.20	.91/.80/.87/.80
5 Class	43626.62	43748.04	.79	.67/.02/.24/.04/.02	.91/.79/.78/.74/.83
6 Class	43555.34	43694.97	.77	.02/.01/.13/.69/.03/.12	.84/.77/.68/.89/.79/.70

BIC = Bayesian Information Criterion, AIC = Akaike Information Criterion

**Fig 2:**
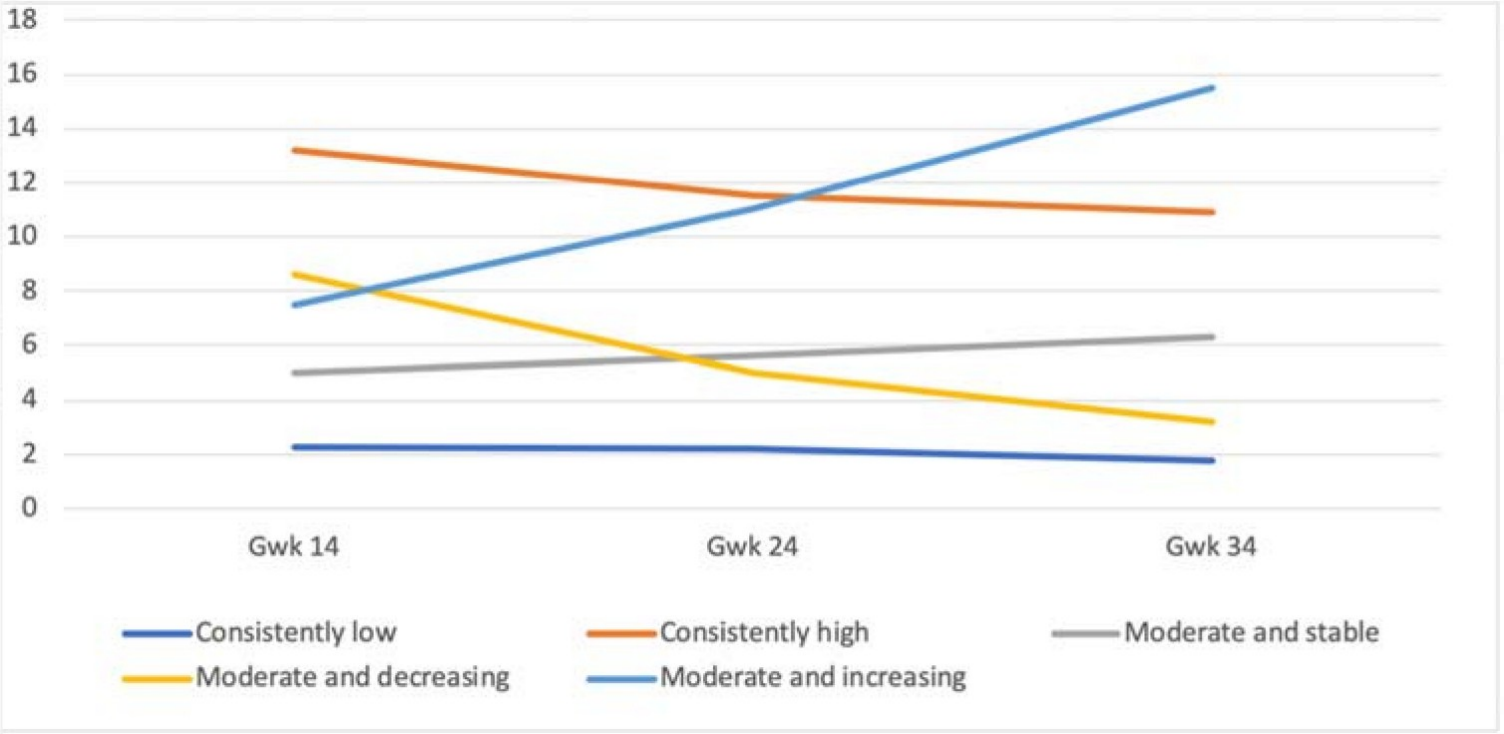
The five-class solution of the trajectories of paternal depressive symptom during pregnancy.

In fathers, the five-group solution yielded similar results as with mothers (Fig 2). The resultant fit indices are presented in Table 3. A large group of fathers with consistently low depressive symptom scores (N = 1475, 71%, M = 2.24; Estimate = –0.30, p < .001) was identified, and a smaller group with consistently high symptom scores (N = 66, 3%, M = 13.18; Estimate = –1.43, p < .001). Three other groups were divided into those with moderate and stable (N = 307, 15%, M = 5.03; Estimate = 0.90, p < .001), moderate and decreasing (N = 185; 9%; M = 8.61; Estimate = –0.29, p < .001), and moderate and increasing symptom scores (N = 42; 2%; M = 7.50; Estimate = 3.85, p < .001). Although this solution did not reach as optimal a fit based on statistical indices as the other solutions, it was chosen because it was replicated as completely similar with the non-imputed data.

**Table 3:** The growth mixture model indices: Trajectories of paternal depressive symptoms during pregnancy.

	AIC	BIC	Entropy	Class Proportions	Average Latent Class Posterior Probabilities
EPDS					
1 Class	24975.14	25020.24	1.000	1.000	1.000
2 Class	24511.83	24573.84	.88	.92/.09	.98/.88
3 Class	24304.56	24383.51	.87	.88/.04/.08	.96/.89/.83
4 Class	24161.38	24257.22	.84	.02/.79/.05/.14	.88/.94/.82/.80
5 Class	24013.63	24126.38	.81	.15/.71/.03/.02/.09	.76/.92/.84/.90/.74
6 Class	23926.68	24056.34	.81	.17/.68/.03/.01/.08/.03	.76/.91/.83/.95/.73/.87

BIC = Bayesian Information Criterion, AIC = Akaike Information Criterion

**Fig 3:**
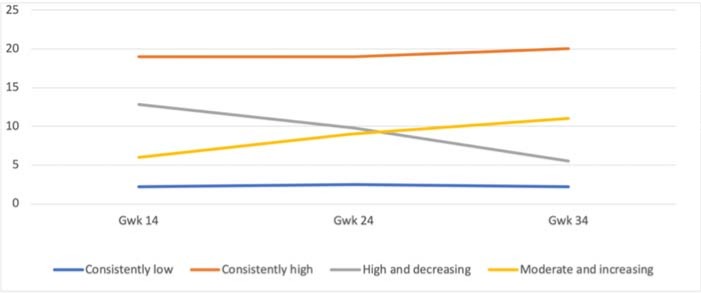
The four-class solution of the trajectories of maternal anxiety symptoms during pregnancy.

For the maternal anxiety symptom trajectories, the four-class solution was chosen, as the five-class solution did not provide any additional information on the course of symptoms (Fig 3). The resultant fit indices are presented in Table 4. The solution comprised one large group with consistently low symptoms (N = 2718, 85%; M = 2.20; Estimate– 0.08, p = .06) and three smaller groups with high or fluctuating symptoms. The smaller groups included mothers with consistently high symptom scores throughout the pregnancy (N = 27; 1%; M = 19.25, Estimate = 0.36, p = 0.64), mothers with high and decreasing symptom scores (N = 213; 7%, M = 12.53; Estimate = –3.49, p < .001) and mothers with moderate and increasing symptom scores (N = 243; 7%, M = 6.37; Estimate = 2.72, p < .001).

**Table 4:** The growth mixture model indices: Trajectories of maternal anxiety symptoms during pregnancy.

	AIC	BIC	Entropy	Class Proportions	Average Latent Class Posterior Probabilities
SCL					
1 Class	43839.20	43887.77	1.000	1.000	1.000
2 Class	42779.04	42845.82	.93	.08/.92	.90/.99
3 Class	42102.32	42187.31	.92	.06/.07/.88	.88/.87/.98
4 Class	41809.45	41912.66	.92	.85/.01/.07/.07	.97/.96/.86/.88
5 Class	41518.84	41640.26	.91	.82/.01/.02/.09/.07	.97/.96/.87/.85/.83

BIC = Bayesian Information Criterion, AIC = Akaike Information Criterion

**Fig 4:**
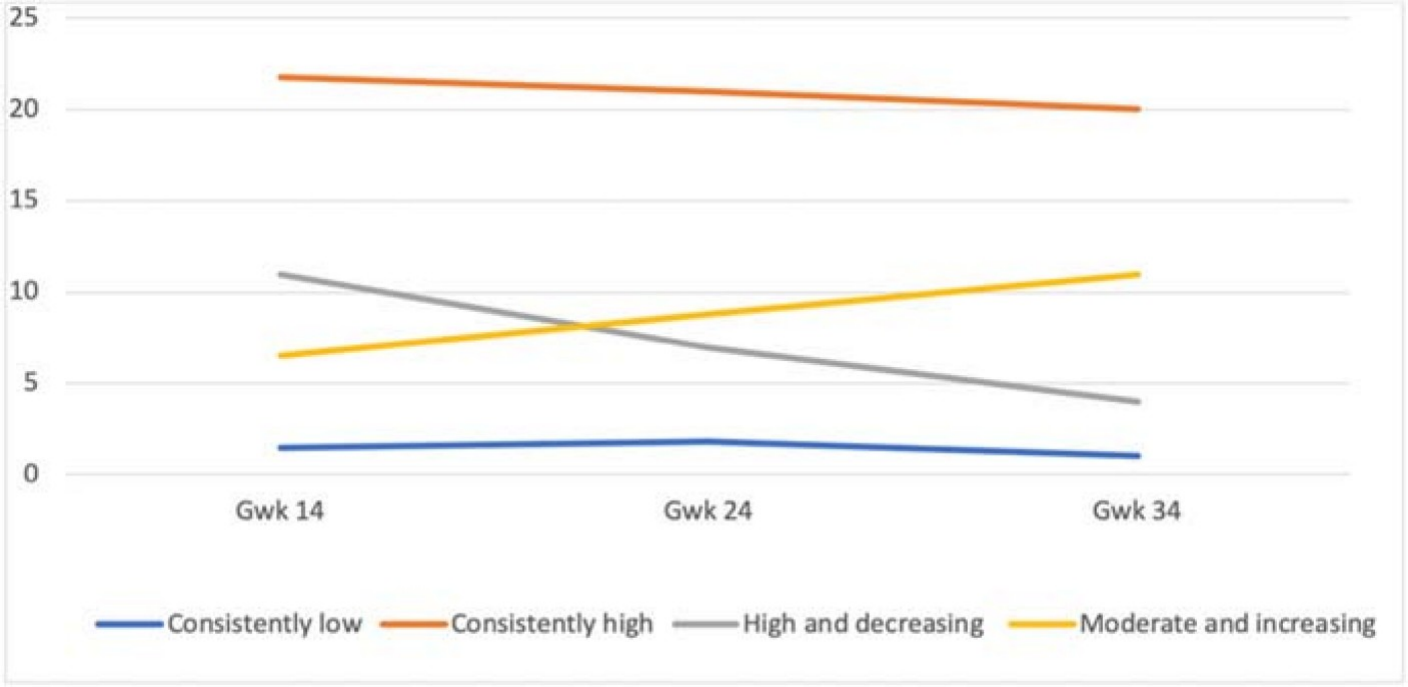
The four-class solution of the trajectories of paternal anxiety symptoms during pregnancy.

Similarly, to mothers, the five-class solution for the fathers did not provide any additional information on the course of anxiety symptoms, and the four-class solution was thus chosen (Fig 4). The resultant fit indices are presented in Table 5. One large group emerged with a consistently low symptom level (N = 1843; 89%, M = 1.57; Estimate = –0.17, p < .001). Further, a very small group with consistently high symptom counts throughout the pregnancy (N = 16; 1%; M = 22.06; Estimate = –1.30, p = .086) was identified. However, this group consisted of only 16 fathers. Two other small groups had either high and decreasing (N = 108; 5%; M = 10.58, Estimate = –3.45, p < .001) or moderate and increasing symptom levels (N = 109; 5%; M = 6.19, Estimate = 2.64, p < .001).

**Table 5:** The growth mixture model indices: Trajectories of paternal anxiety symptoms during pregnancy.

	AIC	BIC	Entropy	Class Proportions	Average Latent Class Posterior Probabilities
SCL					
1 Class	25045.32	25090.42	1.000	1.000	1.000
2 Class	24101.10	24163.12	.96	.95/.05	.99/.93
3 Class	23685.54	23764.48	.95	.91/.04/.05	.99/.88/.88
4 Class	23296.80	23392.65	.94	.01/.89/.05/.05	1.00/.98/.85/.91
5 Class	23055.91	23168.67	.91	.03/.04/.01/.84/.09	.83/.91/.99/.97/.80

BIC = Bayesian Information Criterion, AIC = Akaike Information Criterion

### The associations between symptom trajectories and sociodemographic factors

The trajectories of maternal prenatal depressive symptoms differed in parity, household (living together) and smoking during pregnancy, and in educational level as well as in the use of SSRI medication at 34 gestational weeks in both parents. The mothers in the group of consistently low depressive symptoms included a lower proportion of mothers with lower education compared with the other depressive symptom groups mentioned above (χ^2^ (12) = 60.58, p < .001). In addition, the fathers who had consistently high depressive symptoms had more often a lower educational level than the fathers in the other groups (χ^2^ (12) = 23.36, p = .03). In addition, higher numbers of both mothers (3^rd^ trimester) and fathers (1^st^ trimester) with SSRI medication were observed in the groups of consistently high depressive symptoms and in the group of moderate and increasing depressive symptoms (Mothers: χ^2^ (3) = 59.1, p < .001, Fathers: χ^2^ (3) = 35.8, p < .001) compared to parents in other groups. In addition, mothers in the group of consistently low depressive symptoms were more often primiparous (χ^2^ (4) = 12.91, p = .012), non-smoking at the third trimester (χ^2^ (12) = 30.31, p = .001), and were more often living together with the father of the child (χ^2^ (4) = 34.51, p< .001) compared to the other trajectory groups.

The trajectory groups of prenatal anxiety symptoms differed in parity, household (living together) and smoking during pregnancy in mothers and in age, educational level, and in the use of SSRI medication at 34 gestational weeks in both parents. Mothers with consistently low levels of anxiety were, on average, older (F (3, 3197) = 11.28, p < .001), were living more often with the father of their child (χ^2^ (3) = 30.22, p < .001) and smoked less often during the third pregnancy trimester (χ^2^ (3) = 58.9, p = < .001) than the mothers in all the other groups. In addition, mothers in the groups with increasing or consistently low symptoms had higher educational levels than mothers in both the groups of consistently high as well as moderate and decreasing anxiety (χ^2^ (9) = 52.58, p < .001). Moreover, mothers with consistently high symptom levels were more often multiparous (χ^2^ (3) = 12.24, p < .01) compared to the mothers with increasing and decreasing symptoms. Fathers with decreasing anxiety symptoms were younger than the fathers in the other groups (F (3, 2066) = 5.02, p < .01). In addition, the groups with either consistently high, increasing or decreasing symptoms comprised of more fathers with lower education than the group with consistently low symptoms (χ^2^ (9) = 45.60, p < .001). Furthermore, higher numbers of both mothers (26%, χ^2^ (3) = 85.3, p < .001) and fathers (21%, χ^2^ (3) = 35.1, p < .001) using SSRIs during pregnancy were seen in the group of consistently high symptoms compared to parents in all the other trajectory groups of anxiety. Descriptive values of the associations between trajectories and all background variables are shown in Table 6.

**Table 6:** Associations between different trajectories and background factors.

Mothers n = 3202	EPDS Low and stable (n = 2157)	EPDS High and stable (n = 67)	EPDS Moderate (n = 773)	EPDS Decreasing (n = 131)	EPDS Increasing (n = 72)	SCL Low and stable (n = 2718)	SCL High (n = 27)	SCL High and decreasing (n = 243)	SCL Moderate and increasing (n = 213)	Whole sample
Age	31.1 (4.4)	30.4 (5.1)	30.9 (4.6)	30.7 (4.7)	30.5 (4.9)	31.10 (4.9)	28.4 (5.3)	30.2 (4.6)	29.7 (5.0)	30.9 (4.6)
Education										
Secondary school or less	2.3%	11.7%	3.1%	3.7%	6.2%	2.3%	12.5%	6.2%	6.9%	3.0%
High school/vocational	31.7%	45.0%	36.9%	41.1%	41.1%	33.5%	50.0%	37.6%	46.0%	34.7%
University/Polytechnic	61.3%	38.3%	52.3%	48.8%	48.8%	59.8%	33.3%	52.2%	44.1%	58.0%
Doctoral/Licensiate	4.6%	5.0%	7.7%	3.9%	3.9%	4.4%	4.2%	4.0%	3.0%	4.3%
Nulliparity	53.9%	45.8%	46.9%	46.6%	50.0%	50.8%	41.7%	53.4%	62.9%	51.7%
SSRI-medication at 3rd tri	1.6%	14.6%	4.8%	5.1%	11.3%	1.9%	26.1%	8.3%	8.9%	3.1%
Smoking at 3rd tri	3.98%%	17.3%	9.6%	16.0%	9.7%	4.6%	18.5%	9.9%	13.1%	5.7%
Living together with father	97%	94%	95%	90%	92%	97%	89%	92%	92%	96%
Fathers n = 2076	EPDS Low and stable (n = 1475)	EPDS High and stable (n = 66)	EPDS Moderate (n = 307)	EPDS Decreasing (n = 185)	EPDS Increasing (n = 42)	SCL Low and stable (n = 1470)	SCL High (n = 16)	SCL High and decreasing (n = 108)	SCL Moderate and increasing (n = 109)	Whole sample
Age	32.6 (5.3)	33.1 (5.3)	32.6 (5.4)	32.4(6.2)	32.5(5.3)	32.7 (5.3)	31.6 (5.1)	30.9 (5.0)	32.0 (5.7)	32.6 (5.3)
Education										
Secondary school or less	3.8%	9.5%	7.2%	6.2%	10.8%	3.9%	14.3%	10.5%	13.1%	4.8%
High school/vocational	42.6%	50.8%	46.8%	44.9%	40.5%	42.8%	64.3%	44.8%	52.3%	43.6%
University/Polytechnic	50.5%	39.7%	44.7%	46.1%	48.6%	50.2%	21.4%	44.8%	34.6%	48.9%
Doctoral/Licensiate	3.2%	0.0%	1.4%	2.8%	0.0%	3.1%	0.0%	0.0%	0.0%	2.7%
SSRI -medication at 1st tri	1.3%	10.9%	2.8%	2.27%	8.1%	1.6%	21.4%	3.8%	5.8%	2.1%

### Congruence between depressive and anxiety symptom trajectories and between maternal and paternal symptom trajectories

The congruences between the trajectories of depressive and anxiety symptoms was studied using cross-tabulation, and the results are presented in Table 7. Most mothers and fathers with consistently low depressive symptoms belonged to the group of consistently low anxiety symptoms. Furthermore, the congruence between increasing depressive and anxiety symptom trajectories was relatively high. The most incongruent distributions found between the groups of consistently high levels of depressive and anxiety symptoms, both in mothers as well as in fathers.

**Table 7:** Congruence (%) between depressive and anxiety symptom trajectories.

Mothers			
EPDS consistently low		EPDS consistently low	
SCL consistently low	96	SCL consistently low	98
SCL consistently high	0	SCL consistently high	0
SCL moderate, increasing	2	SCL moderate, increasing	1
SCL high, decreasing	2	SCL high, decreasing	1
EPDS consistently high		EPDS consistently high	
SCL consistently low	19	SCL consistently low	28
SCL consistently high	22	SCL consistently high	15
SCL moderate, increasing	24	SCL moderate, increasing	26
SCL high, decreasing	35	SCL high, decreasing	30
EPDS moderate and stable		EPDS moderate and stable	
SCL consistently low	69	SCL consistently low	79
SCL consistently high	1	SCL consistently high	0.5
SCL moderate, increasing	18	SCL moderate, increasing	14
SCL high, decreasing	12	SCL high, decreasing	7
EPDS moderate, decreasing		EPDS moderate, decreasing	
SCL consistently low	52	SCL consistently low	67
SCL consistently high	3	SCL consistently high	1
SCL moderate, increasing	8	SCL moderate, increasing	8
SCL high, decreasing	37	SCL high, decreasing	24
EPDS moderate, increasing		EPDS moderate, increasing	
SCL consistently low	38	SCL consistently low	33
SCL consistently high	3	SCL consistently high	7
SCL moderate, increasing	57	SCL moderate, increasing	55
SCL decreasing	1	SCL high, decreasing	5

Mothers: χ^2^ (12) = 1346.36, p < .001, partial η^2^ = 0.44; Fathers: χ^2^ (12) = 885.22, p < .001, partial η^2^ = 0.43;

The overlap of maternal and paternal trajectories within families was examined using cross tabulation (Tables 8 & 9). Notable congruence between mothers and fathers was found only between the consistently low symptom trajectories, i.e. the most prevalent categories in this study population. In families where mothers reported consistently low levels of depressive or anxiety symptoms throughout the pregnancy, the fathers also reported low levels of depressive or anxiety symptoms throughout the pregnancy. The congruence between mothers and fathers in the other depressive or anxiety symptom trajectory groups was generally low.

**Table 8:** The congruence (%) between maternal and paternal trajectories of depressive symptoms.

Depressive symptoms EPDS	Consistently low Father	Consistently high Father	Moderate Father	Moderate, decreasing Father	Moderate, increasing Father
Consistently low Mother	51	1.9	9.4	5.5	1.1
Consistently high Mother	1.0	0.2	0.3	0.2	0.2
Moderate Mother	15.6	0.8	4	2.3	0.5
Moderate, decreasing Mother	2.0	0.2	0.9	0.5	0.1
Moderate, increasing Mother	1.3	0.1	0.3	0.3	0

χ^2^ (16) = 55.81, p < .001, partial η^2^ = 0.10

**Table 9:** The congruence (%) between maternal and paternal trajectories of anxiety symptoms.

Anxiety symptoms SCL	Consistently low Father	Consistently high Father	Moderate, increasing Father	High, decreasing, Father
Mother Consistently low	77	0.7	4.0	3.9
Mother Consistently high	0.6	0	0	0.1
Mother Moderate increasing	5.9	0	0.7	0.5
Mother High decreasing	5.2	0	0.5	0.7

χ^2^ (9) = 31.80, p < .001, partial η^2^ = 0.10

## Discussion

In the present study, we identified five different trajectories describing the course of prenatal depressive symptoms both in mothers and in fathers. Regarding the prenatal anxiety symptoms, four different trajectories were identified, equally in both parents. The five trajectories of prenatal depressive symptoms included the following groups: consistently low levels of symptoms, consistently high levels of symptoms, moderate levels of symptoms, moderate and decreasing levels of symptoms and moderate and increasing levels of symptoms. Regarding prenatal anxiety symptoms, the four trajectories were: consistently low levels of symptoms, consistently high levels of symptoms, high and decreasing levels of symptoms and moderate and increasing levels of symptoms.

Our findings are in line with previous trajectory studies indicating 3 to 6 prenatal parental psychiatric symptom trajectories, including at least one trajectory of chronically high symptoms and one trajectory of consistently low symptom levels during the transition from pregnancy to early childhood [32,34,37]. Our findings suggest that potentially important variance exists in the timing and persistence of parent-reported depressive and anxiety symptoms, already during the prenatal period. This should be considered when prenatal exposures are studied, and when child outcomes related to both maternal and paternal symptoms are investigated. Our findings also indicate that higher educational level and older age may be associated with consistently low levels of prenatal psychiatric symptoms in both parents. In mothers, consistently low symptom levels were related to primiparity, not smoking at the 3^rd^ trimester, and living together with the father of the child. Consistently elevated symptom levels were related to the use of SSRI medication in both parents. The congruence between the parents’ trajectories inside families as well as between the trajectories of depressive and anxiety symptoms were relatively low suggesting that the trajectories of depressive and anxiety symptoms have partially independent courses within child-expecting families. However, the congruence in consistently low symptom levels between mothers and fathers within families, as well as between anxiety and depressive symptoms, was notable. This could be explained by the preponderance of the low symptom category in this generally healthy population-based cohort sample.

Approximately 85–90% of the parents in the FinnBrain Cohort reported low levels of depressive or anxiety symptoms at any trimester. This is in line with several previous studies showing that most parents in the general population report only minimal or mild symptoms during pregnancy and the postpartum period [32,33,35,36]. Mothers with low levels of depressive or anxiety symptoms were more often living with the father of the child, smoked less frequently, and were expecting their first child more often than mothers with high or changing levels of depressive or anxiety symptoms. While it has been recognized earlier that parental psychiatric symptoms often co-occur with other risk factors, our notion of several known sociodemographic risk factors accumulating especially in certain symptom trajectories deserves further attention. Thus, it may be that not only the chronicity of symptoms but also the affiliated risk factors play an important role when the offspring outcomes are considered.

Only 1–2% of the parents in our sample experienced consistently high levels of depressive or anxiety symptoms throughout the pregnancy. In line with previous studies, these parents were also more likely to have lower education and more previous children, suggesting that, among other risk factors, socioeconomic burden and the demands of everyday life are associated with the emergence of parental psychiatric symptoms not only during the postnatal but also during the prenatal period [65,66]. These parents had also more often SSRI medication, which might reflect the primary severity of their symptoms. Clinically, it would be essential to identify these parents of consistently high levels of depressive or anxiety symptoms, as parents with chronically pronounced psychiatric symptoms reportedly form an evident risk for the offspring as well as for parenting problems and family adversity [5,67,68]. In addition, chronically elevated symptom levels during pregnancy may reflect prolonged (i.e. covering both prenatal and postnatal time periods) psychiatric problems and, based on previous research, strongly predict postpartum depression [69–72]. Further, 24% of mothers and 16% of fathers in our study reported moderate levels (i.e. differential trajectory to those with consistently low symptom levels) of depressive symptoms throughout pregnancy. In addition to identifying the chronically highly depressed and anxious parents, it would also be important to recognize the parents reporting subclinical levels of depressive or anxiety symptoms, especially if they appear as chronic. Recently, it has been emphasized that also parental subclinical, especially chronic, psychiatric symptoms might have negative associations with future child well-being [73–74].

Furthermore, a group of parents reporting decreasing symptom levels was identified, that presented high symptom scores during the first trimester but very low levels of depressive or anxiety symptoms during the third trimester. This decrease in symptoms may reflect a normative psychological adaptation to pregnancy and parenthood, as the first trimester often includes worries and stress due to the uncertainty of the pregnancy, whereas the second trimester is seen as a period of stability, and the third trimester as a period of positive expectations relating to the upcoming birth and parenthood [75]. By contrast, the groups with increasing levels of depressive or anxiety symptoms, including parents with a low level of symptoms in the first trimester but moderate or high levels of symptoms in the third trimester, may reflect a specific psychiatric symptomatology relating to the upcoming birth, parenthood or concerns about the health of the child. High levels of symptoms before birth are reported to have a negative effect on the child through a prenatal programming effect [76–78], or through postnatal factors including more vulnerable parenting behavior transferring from the antenatal period [76,77,79]. Still, only few studies, to date, have considered the longitudinal course of parental symptoms as a factor in pre- and postnatal programming. Further research is needed to explore the mechanisms underlying the different trajectories and to identify how variation in timing and chronicity affect specified child and parent outcomes.

Overall, the mothers in our sample reported more depressive and anxiety symptoms during pregnancy than the fathers, which is in line with previous studies reporting that in general, depressive and anxiety symptoms are more prevalent among females than males both in the prenatal period and overall [9,10]. On the other hand, within the groups of elevated anxiety symptom levels, mothers had lower symptom scores than fathers, suggesting that fathers experiencing psychological distress may represent a relatively severely anxious population. Interestingly, the trajectory solutions of depressive and anxiety symptoms were very similar between mothers and fathers, suggesting that the differences in trajectories are not sex-specific, but rather may depend on other factors that may be more related to the pathophysiologies and the courses of depression/anxiety *per se* [10,32,36].

The findings on the congruence of within-person depressive and anxiety symptom trajectories were only partly supportive of previous reports. Congruence was high between the trajectories of consistently low levels of depressive and anxiety symptoms (the same person is likely to report no symptoms at all), and moderate between the trajectories of increasing symptoms, both in mothers and in fathers. However, the congruence between depressive and anxiety symptoms was low in the groups of both consistently high symptoms and decreasing symptoms suggesting that these trajectories develop independently from one another, in both parents. This supports the previously presented notion that in the context of prenatal and early life stress, parental depressive and anxiety symptoms should be treated independently as stress exposures [47]. The congruence of maternal and paternal depressive and anxiety trajectories within families was also low, suggesting that the trajectories of depressive and anxiety symptoms during the prenatal period develop relatively independently inside the family. However, the congruence between maternal and paternal trajectories was high in the most prevalent categories of consistently low depressive and anxiety symptom levels, which suggests that in the majority of families, neither of the parents experience marked psychological distress. The differential trajectories within families, and the interactions between maternal and paternal symptom courses should be taken into consideration, when related offspring outcomes are investigated.

A few limitations should be pointed out in the present study. First, sample sizes in the groups of high depressive and/or anxiety symptoms levels were relatively low. Drop-outs may partially explain this small number of parents with very high symptoms levels in the study population. On the other hand, imputation in Mplus was used to diminish this bias. Another limitation was that the parents’ symptoms were assessed using self-report questionnaires as opposed to diagnostic interviews that may have yielded more detailed information on mood and anxiety disorder diagnoses. However, the questionnaires employed in this study are widely used and reliable, and have been shown to be robustly in line with clinical assessments [50–53,56]. In the current study, we were not able to examine the mechanisms behind each trajectory, but this remains to be investigated in the follow-up reports after this description of typical symptom courses and their within-family occurrence. Additionally, the necessary next step should be to study the identified trajectories, and more specifically, the significance of timing, intensity and persistence of both maternal and paternal depressive and anxiety symptoms across the antenatal period in relation to offspring well-being and development. Both the independent maternal and paternal effects as well as the interactive effects of spousal symptom levels on child developmental outcomes need further attention.

To the best of our knowledge, this is the first study to evaluate the trajectories of parental depressive and anxiety symptoms during pregnancy in both mothers and in fathers as well as within families, in a large population-based birth cohort setting. Our findings indicate that the longitudinal development of parental depressive and anxiety symptoms during the prenatal period may represent processes independent of parental sex, but more characteristic of the specific psychiatric symptom type. As depressive and anxiety symptoms do not always overlap, they both should be independently targeted in research as well as in clinical work. The simultaneous assessment of both parents during gestation seems warranted also in future research. Importantly, the trajectories found in the present study provide a basis for investigating possible parental and offspring follow-up outcomes in more detail and in a more stratified manner, and for evaluating long-term prognoses of prenatal exposure to either maternal and/or paternal symptoms of depression and anxiety.
